# MObile Technology for Improved Family Planning Services (MOTIF): study protocol for a randomised controlled trial

**DOI:** 10.1186/1745-6215-14-427

**Published:** 2013-12-12

**Authors:** Chris Smith, Uk Vannak, Ly Sokhey, Thoai D Ngo, Judy Gold, Khemrin Khut, Phil Edwards, Tung Rathavy, Caroline Free

**Affiliations:** 1 Marie Stopes International Cambodia (MSIC), Phnom Penh, Cambodia; 2 Evidence, Strategy and Innovation, Health Systems Department, Marie Stopes International (MSI), London, UK; 3 Department of Population Health, London School of Hygiene and Tropical Medicine (LSHTM), London, UK; 4 National Maternal Child Health Center (NMCHC) and Reproductive Health National Programme Manager, Phnom Penh, Cambodia

**Keywords:** Family planning, Post-abortion family planning, Contraception, mHealth, Cambodia

## Abstract

**Background:**

Providing women with contraceptive methods following abortion is important to reduce repeat abortion rates, yet evidence for effective post-abortion family planning interventions are limited. This protocol outlines the evaluation of a mobile phone-based intervention using voice messages to support post-abortion family planning in Cambodia.

**Methods/Design:**

A single blind randomised controlled trial of 500 participants. Clients aged 18 or over, attending for abortion at four Marie Stopes International clinics in Cambodia, owning a mobile phone and not wishing to have a child at the current time are randomised to the mobile phone-based intervention or control (standard care) with a 1:1 allocation ratio.

The intervention comprises a series of six automated voice messages to remind clients about available family planning methods and provide a conduit for additional support. Clients can respond to message prompts to request a phone call from a counsellor, or alternatively to state they have no problems. Clients requesting to talk to a counsellor, or who do not respond to the message prompts, receive a call from a Marie Stopes International Cambodia counsellor who provides individualised advice and support regarding family planning. The duration of the intervention is 3 months. The control group receive existing standard of care without the additional mobile phone-based support.

We hypothesise that the intervention will remind clients about contraceptive methods available, identify problems with side effects early and provide support, and therefore increase use of post-abortion family planning, while reducing discontinuation and unsafe method switching.

Participants are assessed at baseline and at 4 months. The primary outcome measure is use of an effective modern contraceptive method at 4 months post abortion. Secondary outcome measures include contraception use, pregnancy and repeat abortion over the 4-month post-abortion period.

Risk ratios will be used as the measure of effect of the intervention on the outcomes, and these will be estimated with 95% confidence intervals. All analyses will be based on the ‘intention to treat’ principle.

**Discussion:**

This study will provide evidence on the effectiveness of a mobile phone-based intervention using voice messages to support contraception use in a population with limited literacy. Findings could be generalisable to similar populations in different settings.

**Trial registration:**

ClinicalTrials.gov Identifier: NCT01823861

## Background

Globally, an estimated 44 million pregnancies end in abortion each year, of which nearly half are unsafe, resulting in 47,000 maternal deaths [1,2]. The vast majority of unsafe abortions occur in developing countries and account for one in eight maternal deaths. Around 80% of unintended pregnancies in developing countries occur among women who have an unmet need for modern family planning (FP) [1]. Globally, if unmet need for FP were met, an estimated 75% of unsafe abortions could be avoided [3].

The 2010 Cambodia Demographic Health Survey (CDHS) reported that 81% of women of reproductive age did not want any more children, or wished to wait at least 2 years for their next child, but only 35% were using a modern method of contraception [4]. Modern methods include condom, oral contraceptive (OC), injectable, implant, intra-uterine device (IUD) and permanent methods: vasectomy and sterilisation. The low use of contraception might contribute towards the high abortion rate in Cambodia, estimated at 50 per 1,000 women, well above the global average of 28 per 1,000 [5]. Furthermore, among women who have had an abortion, 26% have had more than one.

This highlights the need for more effective interventions to support clients with post-abortion family planning (PAFP).

There has been widespread uptake of mobile phones in low-income countries including Cambodia, with an estimated 19 million mobile subscriptions covering a population of approximately 14 million in 2012 [6]. Marie Stopes International Cambodia (MSIC) client exit surveys have estimated that over 80% of abortion clients own a mobile phone. The use of mobile phones to deliver healthcare (‘mHealth’) has the advantage over face-to-face healthcare delivery in that support can be delivered inexpensively wherever the person is located, when it is needed. This is of particular relevance in Cambodia where the women least likely to use a modern method of contraception are the rural poor [4]. Behaviour change techniques (BCTs) used in face-to-face interventions can be modified for delivery via mobile phones [7].

Previous trials related to smoking cessation and HIV have reported objective evidence of altered health behaviour leading to improved health outcomes [8,9], yet there is more limited evidence related to the use of contraception.

Of the three previous trials in the USA to improve contraceptive use, Kirby (2010) reported no effect on contraception use with phone calls using motivational interviewing techniques [10], and Hou (2010) reported no significant difference in mean numbers of missed pills with simple daily SMS reminders [11]. However, in a larger trial (*n* = 962), Castano (2010) reported that participants receiving daily educational text messages and pill reminders remained more likely to continue at 6 months (OR 1.41, 1.02-1.95) [12].

There is even less evidence for PAFP. No studies have formally reported mHealth PAFP interventions in low-income countries, although the ‘m-assist: Mobile in Medical Abortion’ trial has provisionally reported increased IUD uptake (21% *vs*. 13%) at 7 weeks post abortion with a post-abortion SMS-based intervention in South Africa [13]. Although these study results look promising, to date, the effect of mHealth interventions on PAFP, or on a wider range of contraceptive methods, has not been reliably established.

The MObile Technology for Improved Family Planning (MOTIF) project comprises the development, implementation and evaluation of a mobile phone-based intervention to support PAFP in Cambodia. This protocol outlines our proposed evaluation of the intervention developed.

## Methods/Design

### Study design

**Figure 1:**
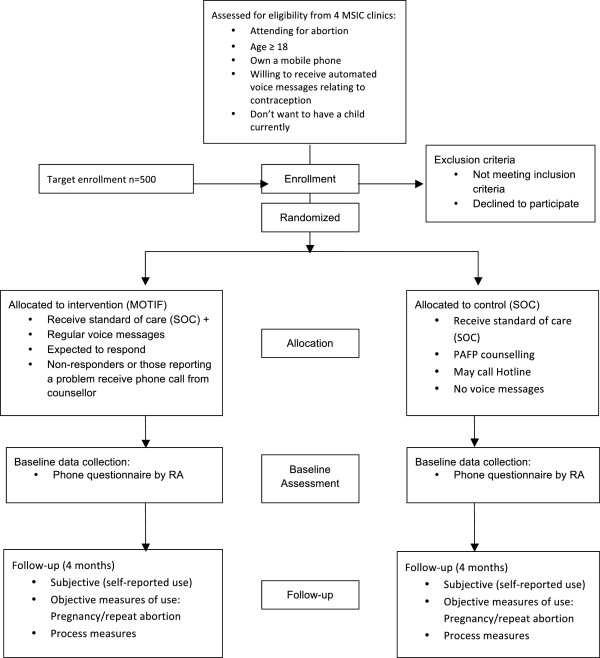
CONSORT diagram of study design.

MOTIF is a multisite single-blind randomised controlled trial (RCT). Participants are randomised to the mobile phone-based intervention (voice messages and follow-up phone calls) or standard of care (SOC)/control (no additional mobile phone-based support) with a 1:1 allocation ratio (see Figure 1).

### Setting and participants

Participants are recruited from four MSIC clinics; two serving predominantly urban populations around Phnom Penh City (Chbar Ambov and Takmao), and two based in provincial towns serving predominantly rural populations (Battambang and Siem Reap). Participants are eligible for the trial if they are attending for induced abortion, aged 18 years or over, own a mobile phone, do not want to have a child at the present time and are willing to receive simple voice messages from MSIC related to contraception. Clients are eligible regardless of whether they have decided to adopt PAFP after their abortion.

Potential trial participants are identified by service providers in the clinics who ask whether they would like to discuss participation in the trial with a research assistant (RA) at the end of the PAFP counselling session. RAs provide further information regarding the study. Given the high rates of illiteracy in Cambodia (literacy in rural areas was 69% according to the 2010 Cambodia DHS), the RA verbally explains the study by reading the Participants Information Sheet [4]. If the client wishes to participate, they sign, or thumbprint, two copies of the consent form. RAs collect baseline data from participants that are recruited. The RA provides a written list of all participants recruited, together with a unique trial identification (ID) number, to the counsellor delivering the intervention. The RA sends only the ID number together with the clinic status (‘urban’ or ‘rural’) of enrolled participants to a project statistician at the London School of Hygiene and Tropical Medicine (LSHTM) via email. Participants are stratified according to urban or rural clinic status and allocated to the intervention or control group using a remote computer-based randomisation programme. Allocation is therefore concealed from RAs working on the trial.

### Intervention

The intervention was developed following a review of the literature, formative research including interviews and focus groups with abortion clients, and with input from clinicians and technology partners in Cambodia [14].

The intervention has a similar basis to the approach used by Lester (2010) in Kenya who hypothesised that a structured mobile phone protocol to keep in touch with patient could improve HIV medication adherence [15]. Detailed description of the intervention development will be reported elsewhere.

**Figure 2:**
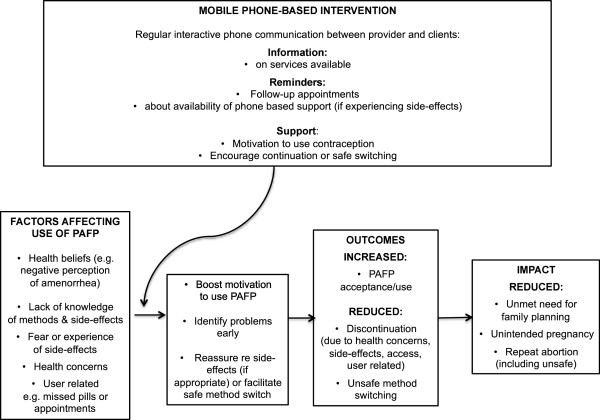
Summary of conceptual framework.

The MOTIF conceptual framework is based on existing literature on the determinants of contraceptive use, links between contraceptive use and fertility, and effective adherence interventions (Figure 2) [16,17].

The MOTIF intervention comprises a series of automated voice messages to participants’ mobile phones over the 3-month period following their abortion, at times of their preference. Clients receive the first message within 1 week of receiving abortion services and then every 2 weeks, with a total of six messages. The messages are designed to remind clients about FP methods available to them and provide a conduit for additional support. A typical message, recorded in the Cambodian (Khmer) language, is as follows:

‘*Hello, this is a voice message from a Marie Stopes counsellor. I hope you are doing fine. Contraceptive methods are an effective and safe way to prevent unplanned pregnancy. I am waiting to provide free and confidential contraceptive support to you. Press 1 if you would like me to call you back to discuss contraception. Press 2 if you are comfortable with using contraception and you do not need me to call you back this time. Press 3 if you would prefer not to receive any messages again*’

Clients who indicate they would like to talk to a counsellor, or who do not respond to the message prompts, receive a call from an MSIC counsellor. The counsellors provide individualised information on contraceptive methods and advice if the client is experiencing side effects from contraception. Clients are advised to use condoms as dual protection from HIV and sexually transmitted infections as appropriate. Follow-up calls to clients are made during preferred times indicated by the client on her registration form. Clients in the intervention arm are also able to call the MOTIF service at any time to request to speak with a counsellor. Clients who opt to receive the OC or injectable can opt in to receive additional reminder messages appropriate to their method (that is, to start a new packet of pills or when to receive a new injection). The sixth and final voice message provides similar information to the first five, but also reminds the client that this will be the last message they will receive.

The MOTIF intervention is delivered by trained counsellors at the MSIC head office in Phnom Penh. Voice messages are scheduled and sent using the open-source software programme ‘Verboice’, developed by InSTEDD (instedd.org). Verboice has functionality with all the mobile phone network operators. MSIC incurs the cost of outgoing communication from the provider to client, and clients incur any costs calling into the service (the cost of a local call). The counsellor records information on the voice messages sent, responses to messages and outcomes of follow-up phone calls.

### Control

Participants in the control group receive the current existing SOC, but not the voice messages or follow-up phone calls. Existing SOC includes: face-to-face post-abortion counselling; a clinic follow-up appointment at 1 or 2 weeks; the clinic phone number and hotline phone number: a toll-free help line for clients staffed by trained counsellors at the MSIC head office.

### Objectives

The objective of the study is to test whether additional regular, structured, interactive mobile phone-based support improves use of PAFP. We hypothesise that the intervention will remind clients about contraceptive methods available, identify problems with side effects early and provide appropriate support, and will boost motivation to use PAFP, while reducing discontinuation and unsafe method switching. Therefore we hypothesise that the intervention will increase use of PAFP compared to clients receiving SOC (control).

### Outcome measures

Baseline characteristics of study participants in both the intervention and control groups will be compared with those of the general clinic population. We will assess recruitment rates, numbers assessed for eligibility compared with numbers enrolled, and completeness of follow-up.

### Primary outcomes

The primary outcome measure is use of an effective modern contraceptive method at 4 months post abortion. We define effective modern methods to be those associated with <10% 12-month pregnancy rates, as commonly used: OC, injectable, implant, IUD, or permanent methods [18]. At 4 months, all trial participants will be contacted by phone to collect self-report data on all outcomes.

Participants are considered ‘users’ or ‘non-users’ of effective modern contraception according to method: implant (participant currently has a sub-dermal implant); IUD (participant currently has IUD inserted); injectable (client has received injection within the previous 3 months); permanent method (client, or husband/partner has had sterilisation or vasectomy procedure); OC (participant reports having taken pill within 24 h of interview or, if on 7-day break, took the last pill according to instructions). Although self-reported data on contraception use are considered less reliable, and prone to social desirability bias, it is the standard approach for contraception research and it will provide data that are comparable to previous studies [19,20].

In order to assess the validity of self-reported data, a reliability study based on approximately 50 participants recruited from the clinics near Phnom Penh will be conducted. Participants who have already provided self-report follow-up who were recruited from Chbar Ambov and Takmao clinics will be contacted in sequential order, and requested to attend the clinic of their choice for face-to-face follow-up for objective measurement on all contraception outcomes. This will include urine pregnancy testing and measures of contraceptive adherence (presence of sub-dermal implant, or documentary evidence of insertion, clinical examination to identify coil threads or documentary evidence of insertion, documentary evidence of injection within the previous 3 months, documentary evidence of sterilisation, pill counts defined as >9% of pills taken since last prescription dispensed).

### Secondary outcomes

Secondary outcome measures include self-reported pregnancy, repeat abortion, contraception use over the 4-month post-abortion period (to estimate point prevalence of contraception use at any given time and contraceptive discontinuation rates), and involvement in any domestic abuse or road traffic accidents (RTA). RTAs are rare, but the only adverse health effect of cell phone use for which there is evidence [21].

### Sample size

Analysis of 2011 MSIC clinic data indicated that the proportion of clients using an effective contraceptive method at 2 weeks post abortion was 44%. The trial will involve the same population so it is reasonable to assume similar PAFP use in the control group. Aggregate demographic and health, and Cambodian, survey data indicate that around 30% of women using hormonal methods and 10% using the coil discontinue within 1 year, many before 3 months of use [22,23]. Contraception use, repeat pregnancy or abortion rates in MSIC clinics after 2 weeks are not known. Based on aggregate data, we anticipate 20% discontinuation from 2-week acceptance, and therefore 35% of clients will be using an effective method at 4 months post abortion.

The trial has been designed to detect an increase of 13% in contraceptive use at 4 months as results from previous mHealth HIV adherence and face-to-face contraception adherence interventions suggest that it is reasonable to anticipate an effect of this size. Canto De Cetina (2001) reported a 26% decrease in injectable discontinuation at 1 year with an intervention providing structured face-to-face counselling *versus* routine information [24]. Lester (2010) reported a 12% increase in self-reported adherence among those receiving the mHealth intervention compared to routine care [15].

In the four trial clinics there were over 1,500 abortions during a 3-month period in 2011, therefore even accounting for refusals and reduced recruitment due to clinic staff time pressure, we believe it will be possible to recruit 500 participants over a 3-month period. A trial of 500 has 80% power to detect a difference in contraceptive use of 35% *vs*. 48% (that is, relative risk 1.4) at the 5% significance level (that is, *P* <0.05). It is not possible to adequately power the study for the rare secondary outcomes of repeat pregnancy and abortion.

### Data collection

At present, around 50% of clients do not return to the clinic for any reason after attending for abortion, therefore all trial participants will be actively followed up by a RA to assess outcome measures. Data collection tools include baseline and follow-up questionnaires: designed in English, translated to Khmer and administered by local RAs fluent in Khmer. The baseline questionnaire contains questions to collect information on contact details, demographics, reproductive history and plans, circumstances of current abortion and mobile phone use.

The follow-up questionnaire contains questions to collect information on changes in demographics, current contraception use, contraception use over the 4-month post-abortion period, and any reported domestic abuse or RTA that could have resulted from mobile phone use. In addition to self-reported measures, clients that attend the clinic for face-to-face follow-up will be offered urine pregnancy testing and objective assessment of contraceptive use by a clinically trained RA.

The follow-up questionnaire assesses births, pregnancies, contraceptive use and discontinuation over a period of time using a similar format to that used in the CDHS.

### Analysis plan

#### ***Analysis of primary and secondary outcomes***

We will report the trial according to the CONSORT standards for reporting RCTs. This is a behavioural intervention unlikely to produce adverse effects, so analysis will be undertaken once the 4-month follow-up has been completed.

Intention-to-treat (ITT) principles will be used for primary outcome analysis; therefore all participants will be analysed according to the arm to which they were randomised. During ITT analysis, participants lost to follow-up, resulting in missing contraceptive use outcome data at 4 months, will be considered non-users.

#### ***Sensitivity and per-protocol analysis***

We will conduct an additional sensitivity analysis including only participants who completed the 4-month follow-up. Per-protocol analysis will be undertaken to assess the impact of the intervention among those who actively participated in the intervention. Participants who respond to three or more of the six voice messages over the intervention period will be considered highly protocol adherent. Participants who respond to between one and three messages will considered less protocol adherent. Those who never responded to a voice message will be considered as never responding and not included in the sensitivity analysis.

#### ***Sub-group analysis***

We will undertake exploratory sub-group analyses to assess evidence for whether the effect of the intervention varies according to age, urban *versus* rural residence, level of education, and socioeconomic status. If statistically significant overall heterogeneity is identified then relative risks and 99% confidence intervals will be estimated.

#### ***Statistical methods***

For the primary outcome and secondary outcomes we will estimate risk ratios with 95% confidence intervals. We will calculate the sensitivity and specificity of self-reported contraception use as compared to objective measurement, and comment on any limitations of the respective methods of data collection. We will undertake Kaplan-Meier survival analysis to compare contraceptive discontinuation rates. Analysis will be conducted using STATA (Table 1) [25].

**Table 1:** Outcome measures and methods of analysis

**Outcome**	**Outcome measure**	**Method of analysis**
**1. Primary**		
Use of an effective modern method of contraception at 4 months	Self-report (binary)	Chi-squared test
	Objective^a^ (binary)	
**2. Secondary**		
Pregnancy	Self-report (binary) (0, 1 or more)	Chi-squared test
	Urine pregnancy test^a^ (binary)	
Repeat abortion	Self-report (binary) (0, 1 or more)	Chi-squared test
Effective modern contraception use over 4-month period	Self-report (binary) (<80%, >80%)	Chi-squared test
Contraceptive discontinuation	Discontinuation after starting contraceptive method (time to event)	Kaplan-Meier survival analysis
Involvement in road traffic accident	Self-report (binary)	Chi-squared test
Domestic abuse	Self-report (binary)	Chi-squared test
**3. Sensitivity and per-protocol analysis**		
Participants that completed follow-up	All outcomes	Chi-squared/T-test/logistic and linear regression
Clustering among participants from one clinic		
Per-protocol analysis		
**4. Sub-group analysis**	All outcomes	
Age		
Urban *versus* rural residence		Logistic/linear regression
Level of education		
Socioeconomic status		

Follow-up at 4 months for all participants will comprise a telephone interview conducted by a research assistant.

^a^Approximately 50 participants from one clinic will be requested to attend for face-to-face follow-up for objective measurement of contraceptive outcomes.

### Additional analysis

#### ***Data arising from the intervention***

We will provide a descriptive analysis of data generated by the intervention to include number of voice message and phone interactions, response to voice messages, and time spent on phone calls, to facilitate description of problems and issues. Additionally, at the end of the trial, the costs of the intervention (training, human resources, phone costs and so on) will be estimated.

#### ***Qualitative interviews***

We will conduct around 15 to 20 qualitative interviews with participants who received the intervention. Participants for interview will be selected purposively to include those who did or did not appear to respond to the intervention: both users and non-users of contraception. The interviews will explore participants’ experience of the intervention, aiming to identify active components of the intervention, and seek recommendations for improvements. Interviews will be recorded and transcribed and a simple thematic analysis undertaken. We will use the findings to inform any adjustments to the intervention after the trial.

#### ***Analysis of long-term follow-up at 12 and 24 months***

The main purpose of taking contraception is to avoid unwanted pregnancy. While it is likely that few participants will report repeat pregnancy and/or abortion at 4 months, differences in these important outcomes may become apparent over a longer period of time. During trial recruitment, participants are given the option to consent for additional follow-up of primary and secondary outcomes at 12 and 24 months. This additional follow-up will be dependent on receiving additional funding and participants will be informed whether this additional follow-up is likely to occur at the end of the study.

### Ethics

The trial is being conducted in accordance with the principles of Good Clinical Practice [26]. Ethical approval for the study protocol was granted by the LSHTM ethics committee, the MSI ethics committee and the Cambodia Human Research Ethics Committee. The MOTIF trial is registered with ClinicalTrials.gov, number: NCT01823861 [27].

All participants provide written consent before enrolling in the trial or commencing the follow-up interview. All client records, written, recorded and transcribed data are stored securely. No names of participants (or others mentioned) or locations will be used in the analysis or report writing. Confidentiality will be maintained by assigning coded identifiers to participant names (with a master list stored separately). Participants are able to withdraw from the study at any point. Any participants raising a personal sexual issue or consequences arising from other people listening to voice messages (for example, an argument or violence) will be linked into appropriate services, either at MSIC, or other local organisations.

Participants are reimbursed to compensate for expenses related to face-to-face clinic follow-up, but not for participation in the trial. Participants are not provided with mobile phones or airtime.

## Discussion

The MOTIF trial will provide rigorous evaluation of a novel mobile phone-based intervention to support PAFP; and will contribute towards the evidence base on mHealth interventions for contraception. The MOTIF trial is unique in a number of ways.

First, to our knowledge this is the first RCT of a mobile phone-based intervention using voice messages to support PAFP. Given the paucity of evidence for effective interventions for PAFP in low-income settings, further research in this area is important. The MOTIF trial has been carefully designed to minimise bias and collect information on important health outcomes, while taking into account the sensitivities of undertaking research with post-abortion clients. Due to the nature of the intervention, the trial can only be single-blind and study participants will be aware of intervention allocation. However, allocation will be concealed to clinicians and RAs working on the trial.

Second, it examines the interventions effect on both users and non-users of contraception, on a range of contraceptive methods over a period of time. Previous mHealth contraception interventions have often been focused on one particular method, often the OC. In addition to the OC, the MOTIF intervention will promote long-acting methods which are associated with less user-failure. We believe that given high rates of dissatisfaction with methods, leading to discontinuation, interventions that promote safe method switching, as well as support for clients experiencing side effects, will lead to increased contraceptive use overall and reduced unintended pregnancy.

Third, to our knowledge, this is one of the first RCTs of a mobile phone-based intervention to support contraception use in a low-income setting. To date, most RCTs of mHealth interventions to support contraception use have been conducted in the USA, the exception being the mAssist trial in South Africa [13]. Other mobile phone-based contraception initiatives that have been launched and scaled up in low-income settings include Mobile for Reproductive Health (m4RH), Cycle Tel, Mobile Alliance for Maternal Action (MAMA), each with different approaches to the intervention and evaluation, but limited evidence of health impacts to date [28-30].

No single trial or initiative will answer the question of whether mHealth interventions are effective in supporting contraception use and we also recognise that mHealth is a dynamic area, with rapid changes in both technology and the techno-literacy of populations; hence, what works or does not work now may not hold true in a few years’ time. The MOTIF trial will provide evidence on the effectiveness of voice messages to support contraception use in populations with limited literacy, and findings could be generalisable to similar populations in different settings. Therefore, in addition to publishing the findings from our statistical analysis we intend to report on our analysis of why the intervention did or did not work, what BCTs appear to be the ‘active components’ of the intervention, technological successes and challenges, and cost and resource implications for scale-up of the intervention. This should ensure that the learning from this study will be of the greatest possible value to other organisations or researchers seeking to develop similar interventions.

## Trial status

Recruiting.

## Abbreviations

BCT: Behaviour change technique; CDHS: Cambodia Demographic and Health Survey; FP: Family planning; ID: Identification; ITT: Intention to treat; IUD: Intra-uterine device; LSHTM: London School of Hygiene and Tropical Medicine; MICE: Multiple impression by chained equations; MOTIF: MObile Technology for Improved Family Planning; MSI: Marie Stopes International; MSIC: Marie Stopes International Cambodia; OC: Oral contraceptive; PAFP: Post-abortion family planning; RA: Research assistant; RCT: Randomised control trial; RTA: Road traffic accident; SMS: Short message service; SOC: Standard of care.

## Competing interests

The authors declare that they have no competing interests.

## Authors’ contributions

CS and CF designed the intervention and the trial. CS drafted the manuscript. UV and LS assisted with formative research to develop the intervention. PE advised on the statistical analysis plan and helped to draft the manuscript. TN, JG, KK and TR participated in the design of the study. CF, TN, JG, KK and TR helped to draft the manuscript. All authors read and approved the final manuscript.
